# Impact of aging on host immune response and survival in melanoma: an analysis of 3 patient cohorts

**DOI:** 10.1186/s12967-016-1026-2

**Published:** 2016-10-19

**Authors:** Sarah A. Weiss, Joseph Han, Farbod Darvishian, Jeremy Tchack, Sung Won Han, Karolina Malecek, Michelle Krogsgaard, Iman Osman, Judy Zhong

**Affiliations:** 1 Department of Medicine, NYU School of Medicine, New York, NY USA; 2 Interdisciplinary Melanoma Cooperative Group, NYU School of Medicine, New York, NY USA; 3 NYU School of Medicine, New York, NY USA; 4 Department of Pathology, NYU School of Medicine, New York, NY USA; 5 Ronald O. Perelman Department of Dermatology, NYU School of Medicine, New York, NY USA; 6 Division of Biostatistics, Department of Population Health, NYU School of Medicine, 650 First Ave., Room 512, New York, NY 10016 USA; 7 School of Industrial Management Engineering, Korea University, Seoul, South Korea; 8 American Express, New York, NY USA

**Keywords:** Age, Elderly, Melanoma, Host immune response, Tumor infiltrating lymphocytes, SEER, TCGA

## Abstract

**Background:**

Age has been reported as an independent prognostic factor for melanoma-specific survival (MSS). We tested the hypothesis that age impacts the host anti-tumor immune response, accounting for age-specific survival outcomes in three unique melanoma patient cohorts.

**Methods:**

We queried the U.S. population-based Surveillance, Epidemiology, and End Results Program (SEER), the prospective tertiary care hospital-based Interdisciplinary Melanoma Cooperative Group (IMCG) biorepository, and the Cancer Genome Atlas (TCGA) biospecimen database to test the association of patient age at time of melanoma diagnosis with clinicopathologic features and survival outcomes. Age groups were defined as ≤45 (young), 46–65 (intermediate), and >65 (older). Each age group in the IMCG and TCGA cohorts was stratified by tumor infiltrating lymphocyte (TIL) measurements and tested for association with MSS. Differential expression of 594 immunoregulatory genes was assessed in a subset of primary melanomas in the IMCG and TCGA cohorts using an integrative pathway analysis.

**Results:**

We analyzed 304, 476 (SEER), 1241 (IMCG), and 292 (TCGA) patients. Increasing age at melanoma diagnosis in both the SEER and IMCG cohorts demonstrated a positive correlation with tumor thickness, ulceration, stage, and mortality, however age in the TCGA cohort did not correlate with mortality. Older age was associated with shorter MSS in all three cohorts. When the young age group in both the IMCG and TCGA cohorts was stratified by TIL status, there were no differences in MSS. However, older IMCG patients with brisk TILs and intermediate aged TCGA patients with high lymphocyte scores (3–6) had improved MSS. Gene expression analysis revealed top pathways (T cell trafficking, communication, and differentiation) and top upstream regulators (CD3, CD28, IFNG, and STAT3) that significantly changed with age in 84 IMCG and 43 TCGA primary melanomas.

**Conclusions:**

Older age at time of melanoma diagnosis is associated with shorter MSS, however age’s association with clinicopathologic features is dependent upon specific characteristics of the study population. TIL as a read-out of the host immune response may have greater prognostic impact in patients older than age 45. Recognition of age-related factors negatively impacting host immune responses may provide new insights into therapeutic strategies for the elderly.

**Electronic supplementary material:**

The online version of this article (doi:10.1186/s12967-016-1026-2) contains supplementary material, which is available to authorized users.

## Background

Age is an important prognostic factor in cutaneous melanoma, which commonly arises in the elderly [1–3]. The median age at initial melanoma diagnosis is 63 and the highest percentage of melanoma-related deaths occur in patients aged 75–84 [4]. Differences in the natural history of melanoma between younger and older patients have been attributed to reduction in naïve T cells, decreased T cell functionality due to loss of co-stimulatory molecules, T cell exhaustion, and reduction in pro-inflammatory cytokine secretion [5, 6]. Tumor infiltrating lymphocytes (TIL) are believed to be a partial surrogate marker of the host anti-tumor immune response and are also thought to confer prognostic significance in melanoma. However, immunologic metrics have yet to be included in the melanoma American Joint Committee on Cancer (AJCC) staging system [7–11]. It is unclear whether age’s impact on the host immune response is reflected by TIL measurements.

There are several unanswered questions regarding the impact of age on melanoma prognosis. It is unknown whether melanomas of the elderly embody a distinct clinical and biologic entity compared to melanomas in younger patients [12]. Understanding the interplay between age, the host immune response, and the tumor immune microenvironment is especially critical as melanoma is increasing in incidence and U.S. demographics are shifting to a larger aging population. Therefore, the diagnosis and treatment of melanoma patients, particularly at advanced ages and stages, represent both a public health issue and an economic burden [13, 14].

The primary objective of this study is to analyze and dissect the impact of age at time of melanoma diagnosis on clinicopathologic features, the anti-tumor immune response as measured by TILs, and melanoma-specific survival (MSS) by examining three unique melanoma patient cohorts: the U.S. Surveillance, Epidemiology, and End Results Program (SEER), New York University’s (NYU) Interdisciplinary Melanoma Cooperative Group (IMCG) biorepository database, and the Cancer Genome Atlas (TCGA) biospecimen database. Secondly, we aim to identify the functional impact of aging on the host immune response by analyzing differential expression of immunoregulatory genes with aging in the IMCG and TCGA cohorts.

The rationale for analyzing three distinct cohorts is to identify whether conclusions drawn from smaller, more specific patient cohorts like IMCG and TCGA can be extrapolated as representative of the general population, as embodied by SEER. This exploration is particularly crucial given the unique features provided by the IMCG and TCGA databases that are not included in the SEER database such as TIL status, genetic sequencing, and gene expression data. Several publications analyze SEER for melanoma-specific outcomes related to age [3, 15–17] however these studies focus only on limited time frames rather than on the entirety of available SEER data as we provide here. To our knowledge, no studies currently approach age from the angle of a multi-cohort perspective. Clinical and research assumptions are commonly based on data from these cohorts, although newer databases like TCGA contain a wealth of genomic information not yet tested against existing population-based data (SEER) or tertiary care hospital-based (IMCG) data. Understanding how to contextualize data on both aging and the immune response from each cohort is critically important to correctly interpret and apply it in the appropriate clinical setting.

## Methods

### Study populations

#### SEER

SEER is a U.S. population-based, publicly-available database sponsored by the National Cancer Institute (NCI) that records cancer statistics among specific demographic registries representing 28 % of the U.S. population. We queried the SEER database for stage I–IV melanoma patients diagnosed from 1973 to 2012. SEER*Stat Version 8.2.1 (NCI, Bethesda, MD) was used to identify all patients diagnosed with invasive melanoma based on the International Classification of Diseases for Oncology, Third Edition melanoma codes (M8720-8790). Available patient information includes age, gender, year of diagnosis, race, survival in months, and vital status. Available pathologic information includes primary melanoma site, thickness, presence of ulceration or mitoses, and stage at diagnosis, but not TIL status [18, 19]. There is no gene expression data available for the SEER cohort.

#### IMCG

The IMCG database is a tertiary care hospital-based melanoma biorepository at NYU Langone Medical Center. The IMCG protocol is approved by NYU’s institutional review board and authorizes research use of patient tumor biospecimens and blood samples and requires prospective recording of all demographic, clinical, and pathologic patient information, with comprehensive, protocol-driven follow-up. We queried the IMCG database to identify patients with primary cutaneous melanoma who presented to NYU from 2002 to 2013. Informed consent for use of clinical data and tissue was obtained from all patients at the time of study enrollment.

Standard histopathologic features for all primary melanoma cases were reviewed and determined by the IMCG pathologist including primary tumor thickness, ulceration, mitoses, and histologic subtype. TILs are graded as brisk (present throughout the vertical growth phase (VGP) or infiltrating the entire base of the VGP), non-brisk (present in one or more foci of the VGP), or absent (no lymphocytes are in contact with the VGP but may be present in perivascular or fibrotic areas) [20].

#### TCGA

TCGA is a collaborative effort run through the NCI and National Human Genome Research Institute (NHGRI) that conducts genomic analyses on human tumors to understand the molecular basis of cancer [21]. Patients who underwent surgical resection of a primary or metastatic melanoma were consented for the study if the biospecimen had at least 60 % tumor nuclei present and enough volume of material available to undergo all platforms of genomic analysis, as well as availability of germline DNA. Patients were excluded if they received radiation therapy to the site of the biospecimen or received prior systemic therapy, with the exception of adjuvant interferon alfa administered at least 90 days prior to obtaining the tissue sample [21, 22].

TCGA records clinicopathologic information including age, gender, race, primary tumor thickness, ulceration, mitoses, histologic subtype, stage at diagnoses, and survival outcomes [22]. TILs were measured in primary and metastatic melanoma tissues as a lymphocyte score (LScore), defined as the sum of lymphocyte distribution (0–3) and lymphocyte density (0–3). Lymphocyte distribution was graded as: no lymphocytes present in the tumor tissue (0) and lymphocytes present in less than 25 % (1), 25–50 % (2), and greater than 50 % (3) of the tumor tissue. Lymphocyte density was graded as absent (0), mild (1), moderate (2), and severe (3) [22]. Based on this sum, LScores of 0 or 2–6 are possible.

### Immunoregulatory gene expression analysis

Using the IMCG biorepository, RNA was isolated from macrodissection of 84 formalin-fixed paraffin embedded (FFPE) primary melanoma sections using the Rneasy FFPE Kit (Qiagen, Valencia, CA) per manufacturer protocol [23] and was subjected to quality control measures. Nanostring gene expression analysis was conducted per manufacturer protocol using the NCounter^®^ GX Human Immunology Kit (Nanostring Technologies, Seattle, WA, USA), comprised of 594 immunoregulatory genes [24]. RNA sequencing data for 43 primary melanomas previously published by the TCGA [22] (level 3 normalized data) was also analyzed for the same immunoregulatory genes and for validation of the IMCG gene expression analysis.

### Statistical analysis

Patient age at time of initial melanoma diagnosis was classified as: young (≤45), intermediate (46–65), and older (>65), although no human data has clearly identified age categories that define immune system quality.

Baseline patient characteristics in each cohort were compared within each age category using the Chi square test. Baseline characteristics in each cohort were also tested for overall association with age and TIL measurements (IMCG and TCGA only). Kaplan–Meier curves were generated and compared by the log-rank test to estimate MSS distribution for each age group in each cohort and for TIL status in the IMCG and TCGA cohorts. MSS was calculated as time from initial melanoma diagnosis to time of death due to melanoma. Patients who were alive or died due to other reasons were censored, and their MSS was calculated as time from initial melanoma diagnosis to last follow-up time. For this analysis in the TCGA cohort specifically, MSS was calculated as time from TCGA specimen sampling to the time of death due to melanoma or last follow-up time. Multivariate cox regression models were used to calculate adjusted hazard ratios (HR) and 95 % confidence intervals (CI) of older age groups for MSS for each cohort. Multivariate analysis for MSS included gender and melanoma stage at diagnosis and age groups as categorical variables, which are coded as two dummy variables representing intermediate and older age. Pooled HR was calculated using a fixed-effects model to evaluate the relationship between age categories and MSS [25].

IMCG and TCGA gene expression data were compared amongst the three age groups by analysis of variance (ANOVA). False discovery rate (FDR) was estimated by the Benjamin Hochberg approach to account for multiple testing correction. All analyses were performed with R version 3.1.1. Ingenuity pathway analysis (IPA) software (Qiagen, Redwood City, CA, USA) was used to identify immunologic pathways that change most with aging [26]. The core analysis function was used to determine the top pathways, upstream regulators, and regulatory effects associated with aging in IMCG and TCGA primary melanomas.

## Results

### Older age in SEER is associated with male gender and more advanced melanoma stage

We examined the relationship of age at melanoma diagnosis with patient and tumor characteristics in the SEER database. The age distribution of 304,476 melanoma patients from SEER registries from 1973 to 2012 is: ≤45 (n = 72,976, 24 %), 46–65 (n = 117,158, 38 %), and >65 (n = 114,342, 38 %) (Table 1). Male gender predominates in the intermediate (59 %) and older age (64 %) groups compared to the young age group in which there are more females (58 %) (p < 0.001).

**Table 1:** Clinicopathologic patient characteristics stratified by age for the Surveillance, Epidemiology, and End Results (SEER), Interdisciplinary Melanoma Cooperative Group (IMCG), and The Cancer Genome Atlas (TCGA) melanoma cohorts

Age groups	SEER (n = 304,476)							IMCG (n = 1241)							TCGA (n = 292)						
	≤45		46–65		>65		p	≤45		46–65		>65		p	≤45		46–65		>65		p
	n	%	n	%	n	%		n	%	n	%	n	%		n	%	n	%	n	%	
	72,976	24	117,158	38	114,342	38		308	25	439	35	494	40		71	24	128	44	93	32	
Gender																					
Female	42,087	58	48,459	41	41,247	36	<0.001	172	56	186	42	197	40	<0.001	24	34	48	38	39	42	0.56
Male	30,889	42	68,699	59	73,095	64		136	44	253	58	297	60		47	66	80	62	54	58	
Stage																					
I	22,625	83	46,599	79	42,064	71	<0.001	246	82	332	78	314	66	<0.001	22	40	25	24	12	14	<0.001
II	1860	7	5790	10	10,543	18		29	10	54	13	130	27		8	15	28	26	38	44	
III	2123	8	4148	7	3695	6		24	8	38	9	35	7		20	36	48	45	33	38	
IV	673	2	2357	4	2931	5		NA	NA	NA	NA	NA	NA		5	9	5	5	3	3	
Thickness																					
<1.01	21,557	77	43,882	73	39,167	65	<0.001	210	68	265	60	239	48	<0.001	16	30	19	22	7	9	<0.001
1.01–2	3782	14	8612	14	9220	15		59	19	97	22	111	22		19	36	21	24	11	14	
2.01–4	1633	6	4386	7	6747	11		23	7	48	11	86	17		7	13	16	18	25	32	
>4	983	4	2915	5	5218	9		16	5	29	7	58	12		11	21	32	36	34	44	
Ulceration																					
Absent	25,586	92	53,175	89	50,370	83	<0.001	272	88	374	85	374	76	<0.001	29	66	37	46	35	47	0.07
Present	2243	8	6388	11	10,213	17		36	12	65	15	119	24		15	34	44	54	39	53	
Alive status																					
Alive	63,562	87	90,890	78	59,083	52	<0.001	295	96	394	90	388	79	<0.001	44	62	73	57	49	53	0.37
Died of melanoma	6387	9	12,625	11	15,285	13		13	4	30	7	48	10		26	37	50	39	37	40	
Other	3027	4	13,643	12	39,974	35		0	0	15	3	58	12		1	1	5	4	7	8	

Rates of regional and distant disease extent, and thus stage, rise with aging while localized disease, particularly stage I melanoma, is more common in the younger age groups (p < 0.001). Thicker melanomas staged as T3 (2.01–4 mm) and T4 (>4 mm) are more common with aging, with T4 occurring in 4 % of young vs. 9 % of older patients (p < 0.001). Ulceration is also more common in the older age group (17 %) compared to the young (8 %) and intermediate (11 %) age groups (p < 0.001). Overall, male gender and thicker, more ulcerated primary melanomas, translating to more advanced stage at diagnosis, positively correlate with older age older at time of melanoma diagnosis in SEER patients (Additional file 1: Table S1A).

### IMCG replicates all SEER data and is representative of the general population

We examined the relationship of age at melanoma diagnosis with patient and tumor characteristics in the IMCG database and compared this to the SEER data. The age distribution of 1241 melanoma patients in the IMCG cohort from 2002 to 2013 is: ≤45 (n = 308, 25 %), 46–65 (n = 439, 35 %), and >65 (n = 494, 40 %) (Table 1). Similar to the SEER cohort, more males comprise the intermediate (58 %) and older (60 %) age groups, while the young age group has more females (56 %) (p < 0.001).

Stage I melanoma diagnoses are more common in the young (82 %) and intermediate (78 %) compared to the older age group (66 %) (p < 0.001). Similar to SEER, aging is also associated with thicker melanomas. T1 (<1.01 mm) is most common in young (68 %) patients (p < 0.001), whereas T4 is most common in older (12 %) patients (p < 0.001).

Unfavorable prognostic factors such as ulceration occur in only 12 % of the young compared to 24 % of the older age group (p < 0.001). Nodular melanomas (NM), thought to represent a more aggressive histologic subtype than superficial spreading melanoma (SSM), occur in 27 % of the older compared to only 15 % of the younger age group (p < 0.001). TIL grade is not significantly associated with age (p = 0.166) (Additional file 1: Table S1B). Overall, IMCG replicates SEER population-based data, suggesting that extrapolations made from the IMCG database are applicable to the general population.

### Age in TCGA carries less association with aggressive clinicopathologic features compared to SEER and IMCG

We examined the relationship of age at time of specimen acquisition with patient and tumor characteristics in TCGA and compared this to the SEER and IMCG analyses. The age distribution of 292 melanoma patients in TCGA is: ≤45 (n = 71, 24 %), 46–65 (n = 128, 44 %), and >65 (n = 93, 32 %) (Table 1). Compared to IMCG which focuses on primary melanomas, the TCGA cohort included only 41 primary melanomas and a majority of melanoma metastases (160 regional lymph nodes, 52 regional skin or soft tissue metastases, and 35 distant metastases). Mean tumor thickness for the TCGA primary melanomas TCGA was 4.9 mm [22] compared to 1.65 mm in IMCG. Unlike SEER and IMCG, there were no significant differences in gender among the age groups in TCGA (p = 0.56).

Primary tumor thickness and staging trends by age in TCGA were reflective of SEER and IMCG results. Young TCGA patients were more commonly diagnosed with stage I melanoma (36 %) compared to the older age group (13 %), while T4 melanomas occurred more in older (44 %) versus younger (21 %) patients (p < 0.001). Ulceration status does not differ by age (p = 0.37). Overall, the only clinicopathologic features that significantly associated with aging in the TCGA cohort were increasing primary tumor thickness (p = 0.003) and stage (p < 0.001). LScore did not significantly associate with age (p = 0.16) (Additional file 1: Table S1C).

### Older age predicts worse MSS in all three cohorts

**Fig. 1:**
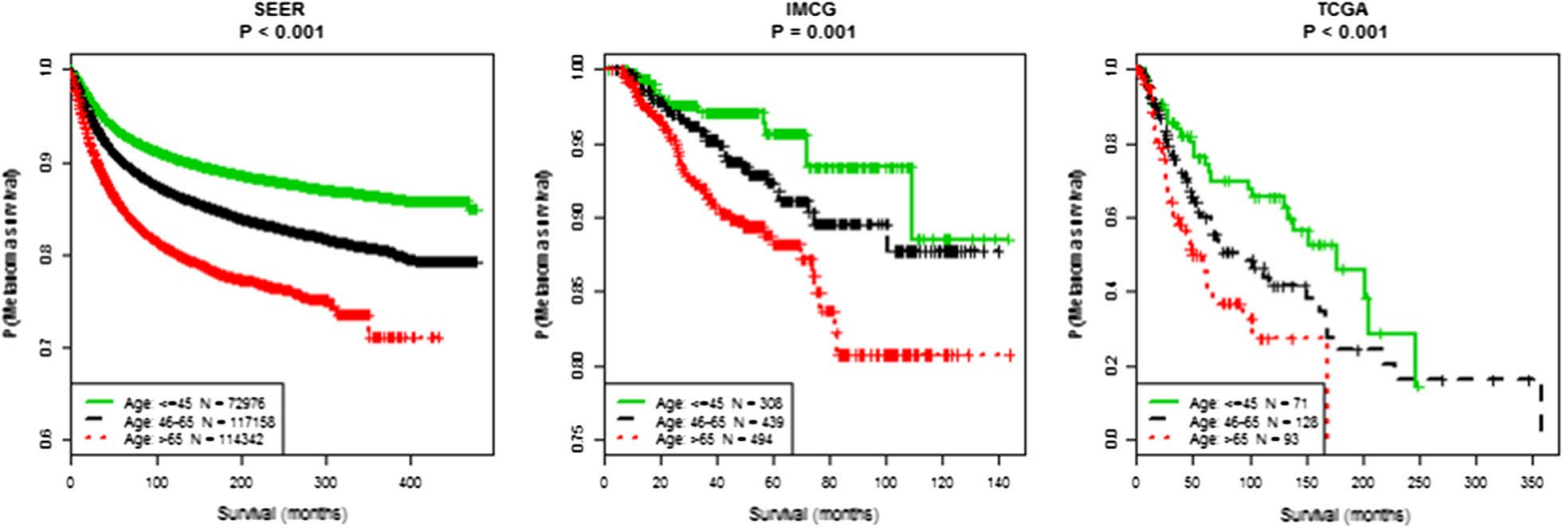
Older age predicts worse melanoma-specific survival in each melanoma cohort

Median follow-up time was 6.25, 4.04, and 3.62 years for SEER, IMCG, and TCGA, respectively. Melanoma-specific mortality rates increase with advancing age at time of melanoma diagnosis in SEER and IMCG (p < 0.001), however there are no significant differences in melanoma-specific mortality rates by age at specimen acquisition in TCGA (p = 0.37) (Table 1) This data is impacted by the varying definitions of age and characteristics required for patient eligibility in each cohort. However, older age predicts worse MSS in all three cohorts (Fig. 1) (SEER p < 0.001, IMCG p = 0.001, TCGA p < 0.001 by log rank test).

**Fig. 2:**
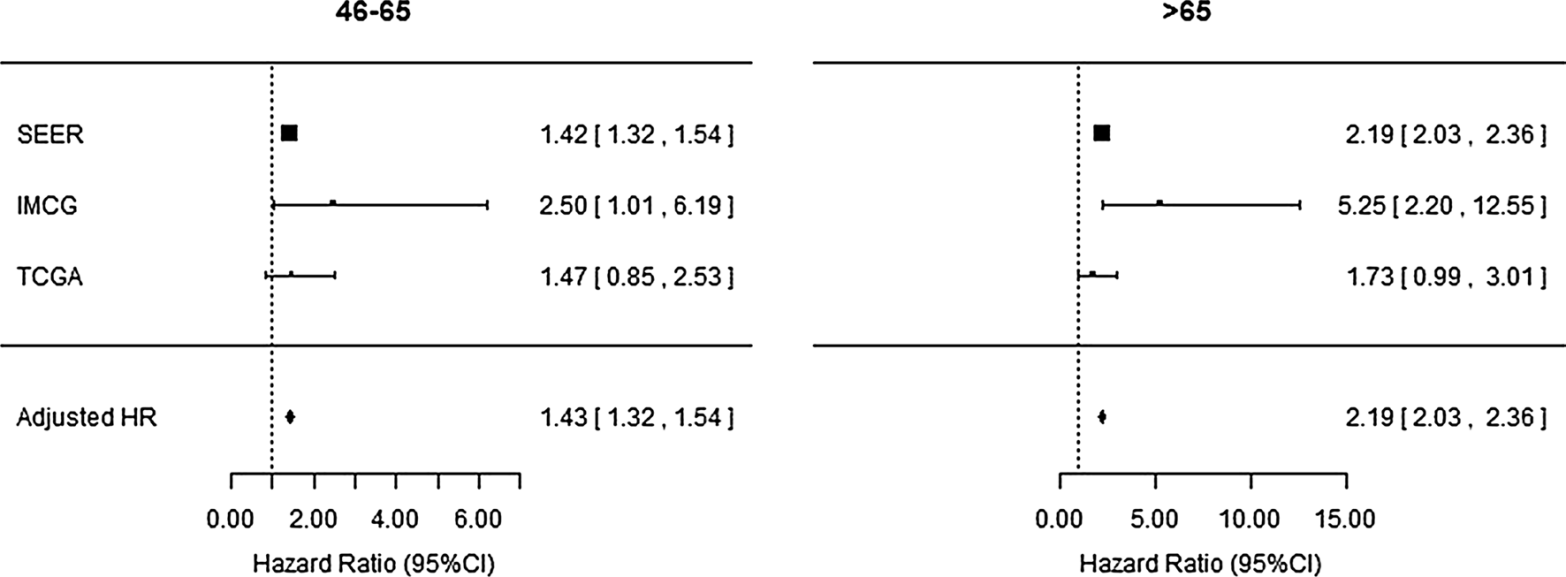
Multivariate analysis and meta-analysis summary demonstrate shorter melanoma-specific survival in middle aged (46–65) and older (>65) patients compared to patients age 45 or less at time of melanoma diagnosis, independent of melanoma stage or gender

Aging corresponds with shorter MSS in each cohort in multivariate analysis, most prominently in SEER and IMCG, independent of gender or stage at diagnosis. Adjusted HRs for MSS in the intermediate compared to the young age group were 1.42 in SEER (95 % CI 1.32–1.54), 2.50 in IMCG (95 % CI 1.01–6.19), and 1.47 in TCGA (95 % CI 0.85–2.53) (Fig. 2). Adjusted HRs for MSS were even higher for older patients in comparison to the young patient reference group: 2.19 in SEER (95 % CI 2.03–2.36), 5.25 in IMCG (95 % CI 2.20–12.55), and 1.73 in TCGA (95 % CI 0.99–3.01) (Fig. 2). Meta-analysis confirms that overall risk of melanoma-specific death is highest in patients older than 65 at time of melanoma diagnosis (adjusted HR 2.19, 95 % CI 2.03–2.36) (Fig. 2).

### TIL intensity positively correlates with MSS in IMCG and TCGA

**Fig. 3:**
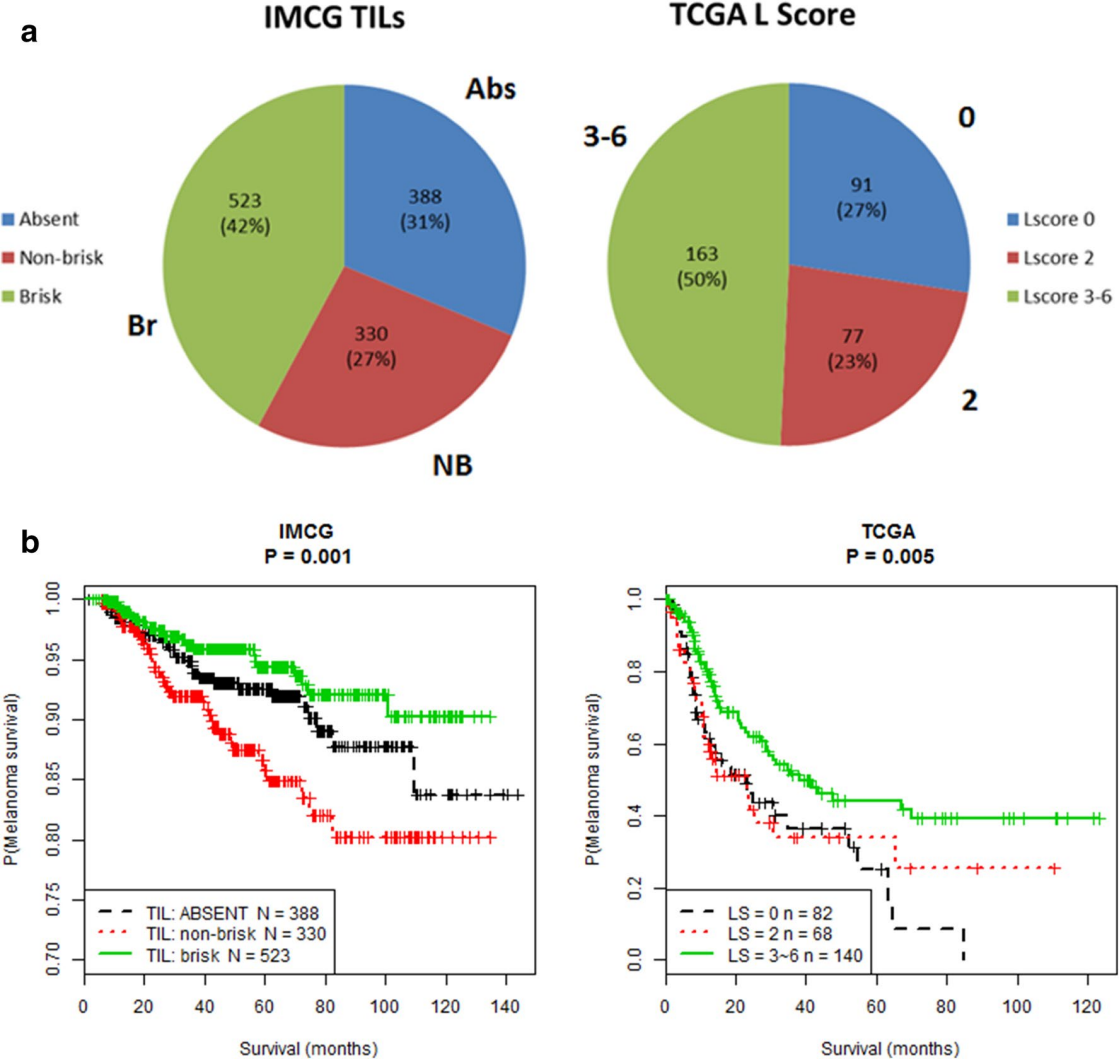
Tumor infiltrating lymphocyte distribution is **a** comparable between the IMCG and TCGA cohorts and **b** is prognostic for melanoma-specific survival

We examined the prognostic impact of TIL grading systems in IMCG and TCGA. In the IMCG cohort, reflective of the SEER population-based data, TIL status derived from primary melanomas was graded as: absent (n = 388, 31 %), non-brisk (n = 330, 27 %), and brisk (n = 523, 42 %) (Fig. 3). Brisk TILs, theoretically representative of a more robust host anti-tumor immune response, predominate in the young (66 %) and intermediate (64 %) age groups compared to older patients (56 %) (p = 0.04). Conversely, the percentage of patients with non-brisk TILs increases from young (34 %) to intermediate (36 %) to older (44 %) age group (p = 0.04). IMCG patients with brisk TIL grade had improved MSS compared to patients with non-brisk and absent TIL grades (p = 0.001).

In TCGA, TILs were scored in both primary and metastatic melanoma tissues: LScore 0 (n = 91, 27 %), 2 (n = 77, 23 %), and 3–6 (n = 163, n = 50 %) (Fig. 3). MSS curves for LScores 3, 4, 5, and 6 clustered together and were therefore grouped. The trend in distribution of LScores amongst TCGA melanomas is very similar to that of TIL grades in IMCG melanomas, despite the differences in tissue sources and age definitions for each cohort. There were no differences in lymphocyte distribution (p = 0.251) lymphocyte density (p = 0.125), or LScores (p = 0.269) amongst the three age groups in TCGA. The percentage of TCGA patients with LScore 0, potentially suggestive of a weaker host immune response, was not significantly different among the young (21 %), intermediate (31 %), or older (29 %) age groups. However, across all age groups, TCGA patients with higher LScores (3–6) had improved MSS compared to patients with lower LScores (0 or 2) (p = 0.005) (Fig. 3). Analysis of the IMCG and TCGA cohorts shows that increased TIL intensity, independent of melanoma cohort, TIL grading system, or tissue type positively correlates with MSS.

### TILs may have greater prognostic value in patients older than age 45

**Fig. 4:**
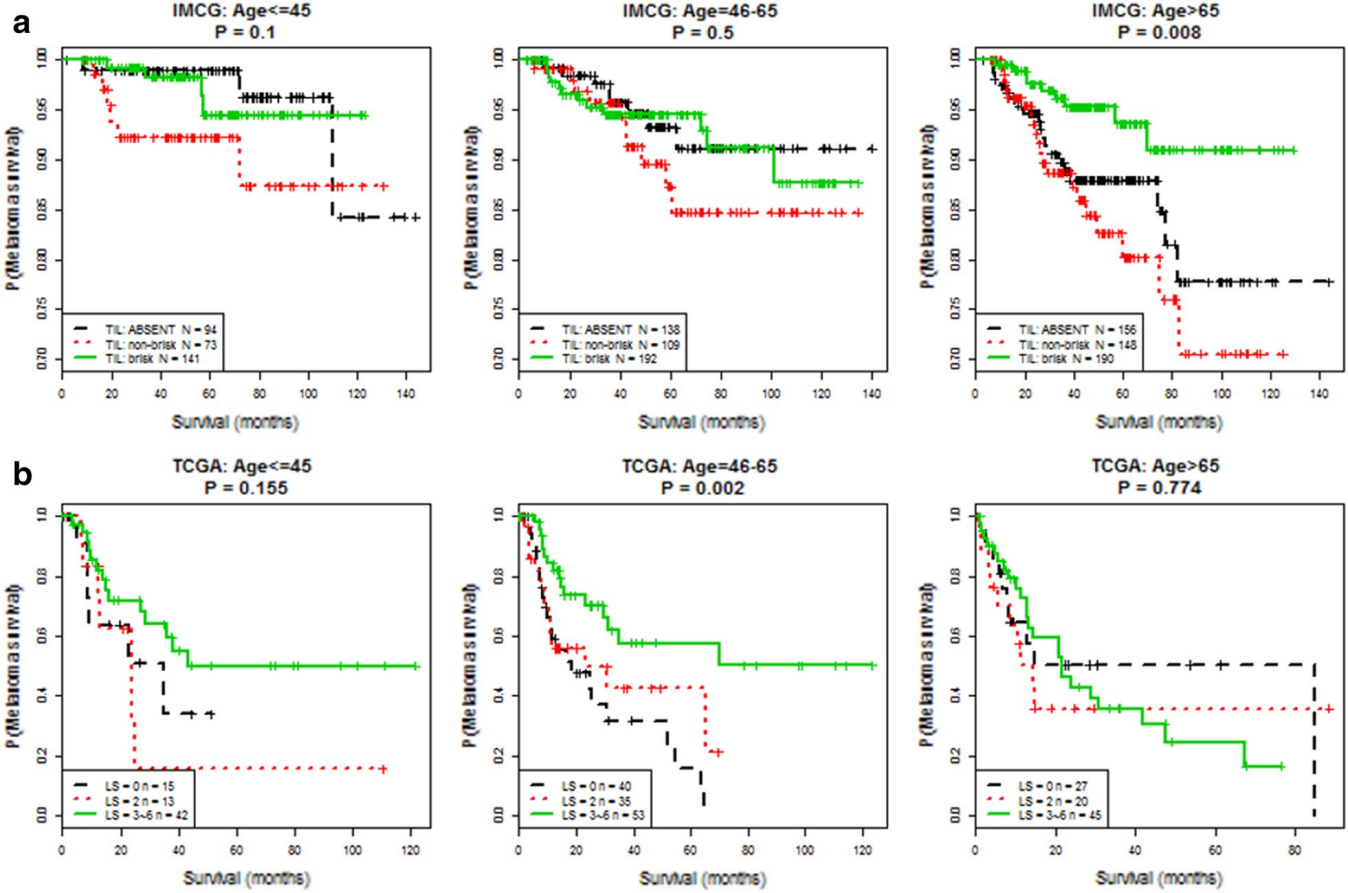
Measures of tumor infiltrating lymphocytes in the **a** IMCG and **b** TCGA cohorts may have more prognostic impact in patients older than 45 years of age

Given that the highest TIL measures (brisk TIL grade and LScore 3–6) are associated with prolonged MSS, we investigated whether the association of robust TILs with MSS persists when TIL status is examined in the context of each individual age group. In IMCG patients, there were no differences in MSS when the young (p = 0.1) or intermediate (p = 0.5) age groups were stratified by TIL grade. However, IMCG patients in the older age group with brisk TILs had improved MSS compared to older patients with non-brisk and absent TILs (p = 0.008) (Fig. 4). In TCGA, LScore stratification in the young (p = 0.155) or older (p = 0.774) age groups did not impact MSS. However, a high LScore (3–6) conferred improved MSS in intermediate aged patients compared to LScore ≤2 (p = 0.005) (Fig. 4). These findings suggest that TIL grading may have greater prognostic impact in patients older than age 45.

### Differential gene expression analysis demonstrates decreased immune cell trafficking in older patients

Differential immunoregulatory gene expression was analyzed among the 3 age groups in IMCG (n = 84) and TCGA (n = 43) primary melanomas. Clinicopathologic characteristics of these melanomas are documented in Additional file 2: Table S2. The most significant differentially expressed genes (p < 0.05) among the young, intermediate, and older age groups are reported for the IMCG and TCGA in Additional file 3: Table S3A and B, respectively. IL13RA1 was the only gene found in both cohorts in this analysis and showed significantly decreased expression with aging.

The top canonical pathways significantly implicated with aging and shared between the cohorts included: (1) crosstalk between dendritic cell and natural killer cells, (2) granulocyte adhesion and diapedesis, (3) T helper cell differentiation, and (4) IL-10 signaling. Additionally, ICOS signaling, CD28 signaling, and role of NFAT in the immune response were also top altered pathways in the IMCG with aging, as was HMGB1 signaling in the TCGA cohort.

Predicted upstream regulators common to both cohorts included CD3, CD28, IFNG, and STAT3. In both the IMCG and TCGA cohorts, there was also diminished cellular function including decreased immune cell trafficking and impaired T cell development.

## Discussion

Our aging analysis of three melanoma cohorts demonstrates with collective agreement that age is an independent prognostic factor for MSS, consistent with several other studies [1, 3, 27]. However, there are inherent differences among the three examined patient populations and we emphasize that drawing broad conclusions from analysis of any one group may be misleading. SEER follows over 300,000 U.S. melanoma patients representative of the general population and is a gold standard from which reliable deductions regarding melanoma demographics, clinicopathologic features, and outcome measures can be made. Our SEER analysis shows that with increasing age, primary melanomas exhibit increased thickness and more unfavorable pathologic features, leading to more advanced stages, increased mortality, and worse MSS, independent of gender and stage at diagnosis. This validates an analysis of 13,581 patients in the AJCC melanoma database in which age was the third most significant prognostic factor for localized melanomas after thickness and ulceration [28–30].

IMCG data identically replicate the benchmark results established by SEER. Therefore, extrapolation of results from our hospital-based IMCG cohort is valid and applicable to the general population that SEER represents, despite the possible referral bias that may be intrinsic to a large, academic, tertiary care medical center. Conversely, the TCGA population is distinct in several ways from SEER and IMCG. TCGA consists of highly pre-selected patients and requires sufficient tissue quantity for genomic studies. Therefore, TCGA is mostly comprised of metastases [22] and the selected primary melanomas are naturally thicker than average. This bias is important in our analysis if we assume that metastatic melanomas differ biologically and immunologically from primary melanomas. Additionally, the age at time of TCGA specimen acquisition often does not match the definition of age at time of primary diagnosis as defined in SEER and IMCG. TCGA sample size is also only 25 % that of the IMCG. These differences aid in explaining why the TCGA age analysis shows no association with gender, ulceration, or mortality rates, unlike SEER and IMCG. The TCGA clearly remains a valuable resource, but interpretation of TCGA data should be applied in the context of that cohort’s specific patient population rather than broadly generalized.

Confounding factors not specifically studied in this investigation should be emphasized as potentially contributing to the worse outcomes seen in elderly patients. Physician bias may occur in which elderly patients are offered less strict clinical surveillance or fewer opportunities for adjuvant therapies. Elderly patients are also less frequently offered SLN biopsies, resulting in a percentage of clinically node negative but pathologically node positive patients, and thus current staging data by age may be underestimated [31]. Additionally, oncologists may hesitate to offer aggressive systemic therapy for advanced melanoma in the elderly [12, 32]. Most immunotherapy clinical trials in melanoma do not prospectively stratify patients by age to assess for differences in outcome, but the few that do show that response to immunotherapy is independent of age. This suggests that age should not be a critical factor in determining patient candidacy for immunotherapies [33–36]. Socioeconomic factors also impact access to health care. Elderly patients may be less able to seek out medical care due to declining functional status, financial considerations, or decreased social support and isolation. Finally, it should be acknowledged that other chronic inflammatory medical conditions and certain viral infections may also correlate with age and could potentially confound our observations. However, this information is not recorded in any of our database cohorts and thus requires a separate analysis.

Melanoma’s escape from an aging, dampened host immune surveillance mechanism is one factor hypothesized to account for decreased MSS in the elderly [6]. This may explain why brisk TIL grade has been proposed to be more common in younger rather than older patients [8]. However, in our datasets, lymphocytic markers did not directly correlate with age on the whole. Although TIL grading is an imperfect tool in gauging host immune function, it is a readily accessible measurement and commonly recorded in melanoma pathology reports, despite lack of consistent clarity on its prognostic impact [9, 11, 20, 37]. In this case, it is possible that use of different age cut-offs may have yielded a significant correlation with TIL groups. Furthermore, the presence of TILs histologically does not necessarily translate into the appropriate functionality. Comparing two different TIL grading systems may also pose a barrier in standardizing our data. TIL grade has been previously studied in primary melanomas and while a potentially subjective measure, has shown high interobserver agreement [10]. Conversely, TCGA LScore has not previously been validated and is employed in a heterogeneous group of tissues consisting of fewer primaries and a majority of metastases. Despite these differences, a high degree of lymphocytic infiltration represented by both brisk TIL grade and LScore 3–6 correlates with improved MSS. In selected patient groups over age 45, the highest TIL measures did correlate with MSS, whereas in the young groups, there were no significant differences in TIL measures with survival. While TILs represent a semi-quantitative measure of lymphocytes, improved markers of the overall tumor immune microenvironment may be even more useful.

Despite limitations in comparing TIL grade and LScore, the proportions of each TIL grade to the corresponding LScore (brisk TILs/LScore 3–6, non-brisk TILs/LScore 2, absent TILs/LScore 0) are strikingly similar. Furthermore, TCGA MSS curves for LScore 0 and 2 overlap until 60 months’ follow-up, mirroring the overlapping IMCG MSS curves for non-brisk and absent TIL grades. The consistent survival distinction between strong lymphocytic infiltrates, represented by brisk TILs/LScore 3–6, and the less intense infiltrates suggests a prognostic cut-off point. TIL classification in young patients did not impact MSS in IMCG or TCGA, which may be partially explained by small sample size of TCGA or because TIL presence tends to have a higher incidence in thin melanomas [7, 38], which are more common in IMCG. Our data highlights that presence of an intense immune infiltrate may carry more prognostic weight in older patient populations, in which more variable levels of or decline in immune function exist. In younger patients (≤45), immune function is more universally intact. Factors other than host anti-tumor immunity may contribute more significantly to prognosis, such as underlying molecular drivers.

To better define the immunologic mechanisms underlying age’s impact on decreased MSS, we analyzed gene expression signatures of primary melanomas stratified by age. Aging was associated with decreased T cell differentiation, activation and migration. The differential gene expression by age was driven by upstream regulators common to each cohort such as CD3, a component of the mature T cell receptor, and CD28, a co-stimulatory molecule. In the IMCG cohort for example, CD28 and CD3 with aging decreased expression of GZMA and GZMB, proteases important in T cell and NK-cell-mediated tumor cell lysis. Overall, with aging there is evidence of a depressed anti-tumor immune response due to T cell dysfunction.

## Conclusion

In summary, we demonstrate through analysis of three distinct melanoma patient cohorts that age at time of melanoma diagnosis is a clear prognostic indicator predicting MSS. SEER and IMCG demonstrate that older age at melanoma diagnosis associates with male gender, advanced stage, and more adverse clinicopathologic features including presence of ulceration, mitoses, and more high-risk histologic subtypes. In contrast, the TCGA cohort is representative of mostly advanced stage patients with thicker melanomas and thus age at time of specimen sampling does not factor into prognosis as significantly. Our study uniquely highlights the similarities and differences of each melanoma cohort on which multiple conclusions regarding prognosis are based. Clinicians should utilize an appropriate melanoma cohort that is specific to their patient population to accurately estimate patient prognosis and to judge age’s impact on the host immune response. Interestingly, TIL status as a measure of the host anti-tumor immune response appears to influence prognosis most in patients older than 45. As TILs are increasingly being considered as an informative prognostic marker, incorporation of age and TIL status as joint prognostic markers may strengthen their value in projecting outcomes compared to either variable alone. Finally, gene expression analysis of each age group has revealed alterations in key regulators of the host immune response with aging. Recognition of age-related factors negatively impacting host immune responses may provide new insights into therapeutic strategies for the elderly.

## Additional files

**Additional file 1: Table S1.** **A** Association of SEER clinicopathologic features with age. **B** Association of IMCG clinicopathologic features with age and tumor infiltrating lymphocytes. **C** Association of TCGA clinicopathologic features with age and lymphocyte score.

**Additional file 2: Table S2.** Clinicopathologic features of 84 IMCG and 43 TCGA primary melanomas in gene expression analysis.

**Additional file 3: Table S3.** **A** Significant gene expression differences with aging in the IMCG primary melanoma cohort (n = 84). **B** Significant gene expression differences with aging in the TCGA primary melanoma cohort (n = 43).

**Additional file 4: Table S4.** Immunoregulatory gene expression of 84 IMCG primary melanomas.

## Abbreviations

AJCC: American Joint Committee on Cancer Staging
ANOVA: analysis of variance
FDR: false discovery rate
FFPE: formalin-fixed paraffin embedded
H&E: hematoxylin and eosin
IMCG: Interdisciplinary Melanoma Cooperative Group
IPA: integrative pathway analysis
LScore: lymphocyte score
MSS: melanoma-specific survival
NM: nodular melanoma
NYU: New York University
SEER: Surveillance, Epidemiology, and End Results Program
SSM: superficial spreading melanoma
TCGA: The Cancer Genome Atlas
TIL: tumor infiltrating lymphocytes
VGP: vertical growth phase
